# Genetic Polymorphism of Angiotensin-Converting Enzyme and Chronic Obstructive Pulmonary Disease Risk: An Updated Meta-Analysis

**DOI:** 10.1155/2016/7636123

**Published:** 2016-10-18

**Authors:** Sang Wook Kang, Su Kang Kim, Joo-Ho Chung, Hee-Jae Jung, Kwan-Il Kim, Jinju Kim, Ju Yeon Ban

**Affiliations:** ^1^Department of Dental Pharmacology, School of Dentistry, Dankook University, Cheonan 31116, Republic of Korea; ^2^Kohwang Medical Research Institute, School of Medicine, Kyung Hee University, Seoul 02447, Republic of Korea; ^3^Division of Allergy and Respiratory System, Department of Korean Internal Medicine, College of Korean Medicine, Kyung Hee University, Seoul 02447, Republic of Korea; ^4^Department of Korean Physiology, College of Pharmacy, Kyung Hee University, Seoul 02447, Republic of Korea

## Abstract

The relationship between polymorphism of the angiotensin I converting enzyme (*ACE*) gene and chronic obstructive pulmonary disease (COPD) has been examined in many previous studies. However, their results were controversial. Therefore, we performed a meta-analysis to evaluate the relationship between the* ACE* gene and the risk of COPD. Fourteen case-control studies were included in this meta-analysis. The pooled *p* value, odds ratio (OR), and 95% confidence interval (95% CI) were used to investigate the strength of the association. The meta-analysis was performed using comprehensive meta-analysis software. Our meta-analysis results revealed that ACE polymorphisms were not related to the risk of COPD (*p* > 0.05 in each model). In further analyses based on ethnicity, we observed an association between insertion/deletion polymorphism of the* ACE* gene and risk of COPD in the Asian population (codominant 2, OR = 3.126, 95% CI = 1.919–5.093, *p* < 0.001; recessive, OR = 3.326, 95% CI = 2.190–5.050, *p* < 0.001) but not in the Caucasian population (*p* > 0.05 in each model). In conclusion, the present meta-analysis indicated that the insertion/deletion polymorphism of the* ACE* gene may be associated with susceptibility to COPD in the Asian population but not in the Caucasian population. However, the results of the present meta-analysis need to be confirmed in a larger sample.

## 1. Introduction

COPD is a serious disease which is characterized by destruction of the lung parenchyma and inflammation of the peripheral airways [1]. COPD is a global health problem due to its high prevalence, morbidity, mortality, and social care cost [2, 3]. The Global Burden of Disease (GBD) study reported that COPD was the sixth leading cause of death in 1990 and the fourth leading cause of death in 2000 [4]. Also, an estimated 4.7 million people die due to COPD [5]. The cause of COPD is multifactorial. Cigarette smoking is known to be the major cause of COPD, but some COPD cases cannot be explained by smoking alone [6]. Environmental exposure such as occupational exposures [7] and indoor biomass fuel burning [8] is closely related to the development of COPD. In addition, many previous studies have reported the association between genetic factors and COPD susceptibility. Previous family and twin studies have shown the role of genetic factors in COPD susceptibility [9]. Recently, some authors have used genetic polymorphisms to explain the genetic contribution to the development of COPD, and several candidate genes such as proteinase-activated receptor-1 [10], plasminogen activator inhibitor-1 [11], and *β*2-adrenergic receptor [12] were reported to have an association with COPD susceptibility.

ACE has been one of the most studied candidate genes [13–16] due to its wide role in the development of various diseases such as systemic lupus erythematosus [17], hypertension, chronic kidney disease [18], and diabetic nephropathy [19]. The* ACE* gene is located on the chromosome 17q23 and it encodes angiotensin-converting enzyme (ACE) which plays a role in converting angiotensin I into angiotensin II. One of the well-known polymorphisms in the* ACE* gene is the insertion/deletion polymorphism, which is 287 bp long and results in three genotypes (II, ID, and DD). The genotypes have been shown to be associated with ACE activity and levels in plasma and tissues [20]. A previous study reported that ACE activity depends on the O2 concentration of the blood and increased ACE levels due to hypoxia could be associated with severe tissue damage [21]. In COPD patients, the ACE activity increased during exacerbation, while it decreased during remission [22]. Inhibition of ACE using an ACE inhibitor could improve the exercise capacity during pulmonary rehabilitation in COPD [23]. ACE has been known to have an association with COPD, and an ACE inhibitor has been used to treat COPD. Thus, many previous studies have attempted to clarify the association between* ACE* polymorphism and COPD susceptibility, but several studies have reported conflicting results. Therefore, the aim of this meta-analysis was to investigate the possible association between* ACE* polymorphism and COPD risk based on all available relevant studies.

## 2. Materials and Methods

### 2.1. Search Strategy

All studies which were published from January 1, 1998, to March 1, 2016, and which examined the association between ACE insertion/deletion polymorphism and COPD risk were carefully searched. Case-control studies were searched in PubMed, Google, Embase, and Korean databases (KISS, KMbase, and RISS) up to March 2015. The following keywords were used: “angiotensin-converting enzyme” or “ACE” AND “polymorphisms” AND “chronic obstructive pulmonary disease or COPD.” Only human studies were selected. Additional studies were identified by a manual search of the reference of the related original studies or review articles. If data or data subsets were published in more than one article, only the publication with the largest sample size was included.

### 2.2. Inclusion and Exclusion Criteria

The inclusion criteria were as follows: (1) the studies should have evaluated the relationship between the ACE insertion/deletion polymorphism and COPD risk; (2) the study design should be a case-control study; and (3) the authors should have provided sufficient data on genotype distributions in the COPD group and the control group to estimate the odds ratio (OR) with a 95% confidence interval (CI). Studies were excluded if the genotype distribution in the control groups deviated from the Hardy-Weinberg equilibrium.

### 2.3. Data Extraction

Two investigators independently extracted data and created an analysis. When the investigators differed in their conclusions, they rechecked the data and reached a consensus through discussion. Data extracted from the selected articles included the first author's name, the year of publication, country of origin, ethnicity of the study population, the number of cases and controls, and the genotype frequency of* ACE* insertion/deletion polymorphism.

### 2.4. Statistical Analysis

Meta-analysis was performed using a comprehensive meta-analysis software program (Biostat Corporation, NJ, USA). The pooled *p* value, OR, and 95% CI were used to investigate the association between the risk of COPD and* ACE* insertion/deletion polymorphism. In the present meta-analysis, we adopted the following genetic models [24, 25]: codominant 1 (I/D genotype versus I/I genotype), codominant 2 (D/D genotype versus I/I genotype), dominant (D/D genotype + I/D genotype versus I/I genotype), and recessive (D/D genotype versus I/D genotype + I/I genotype) models and the allele model (D allele versus I allele). For codominant 1, codominant 2, and dominant models, we used I/I genotype as the reference group. For the recessive model, I/D genotype + I/I genotype was used as the reference group.

Firstly, we assessed the heterogeneity among studies to select the analysis model. The *χ* ^2^-test-based *Q* statistic test and the *I* ^2^ test were applied. The random-effects Mantel-Haenszel method was adopted if the result of the *Q* test was *p* < 0.05 or the *I* ^2^ statistic was >50%, which indicated a statistically significant degree of heterogeneity among studies. Otherwise, the fixed-effects Mantel-Haenszel method was adopted. Publication bias was evaluated by Egger's regression. A *p* value less than 0.05 was considered statistically significant.

## 3. Results

### 3.1. Characteristics of Eligible Studies

Table 1 describes the characteristics of selected studies included in the meta-analysis. Briefly, the meta-analysis in the present study was performed with fourteen articles [13–16, 26–35]. After pooling all data, these 14 articles included 977 patients with COPD and 1,092 control subjects.

### 3.2. Quantitative Synthesis

Among these 14 studies, two studies [15, 26] showed an error in the Hardy-Weinberg equilibrium in the control group (*p* < 0.001 and *p* = 0.004, resp., Table 2). These two studies were excluded from the evaluation of the exact analysis. To conduct risk assessment in all COPD cases and control patients, we assessed the heterogeneity in each model, respectively (codominant, dominant, and recessive models and the allele model). The results of the heterogeneity test for meta-analysis are shown in Table 2. The random-effects method was applied if the result of the *Q* test was *p* < 0.05 or the *I* ^2^ statistic was >50%. Otherwise, the fixed-effects method was adopted. Insertion/deletion polymorphism of the* ACE* gene did not show any significant association with susceptibility to COPD in each model, respectively (*p* > 0.05, Table 2).

**Figure 1:**
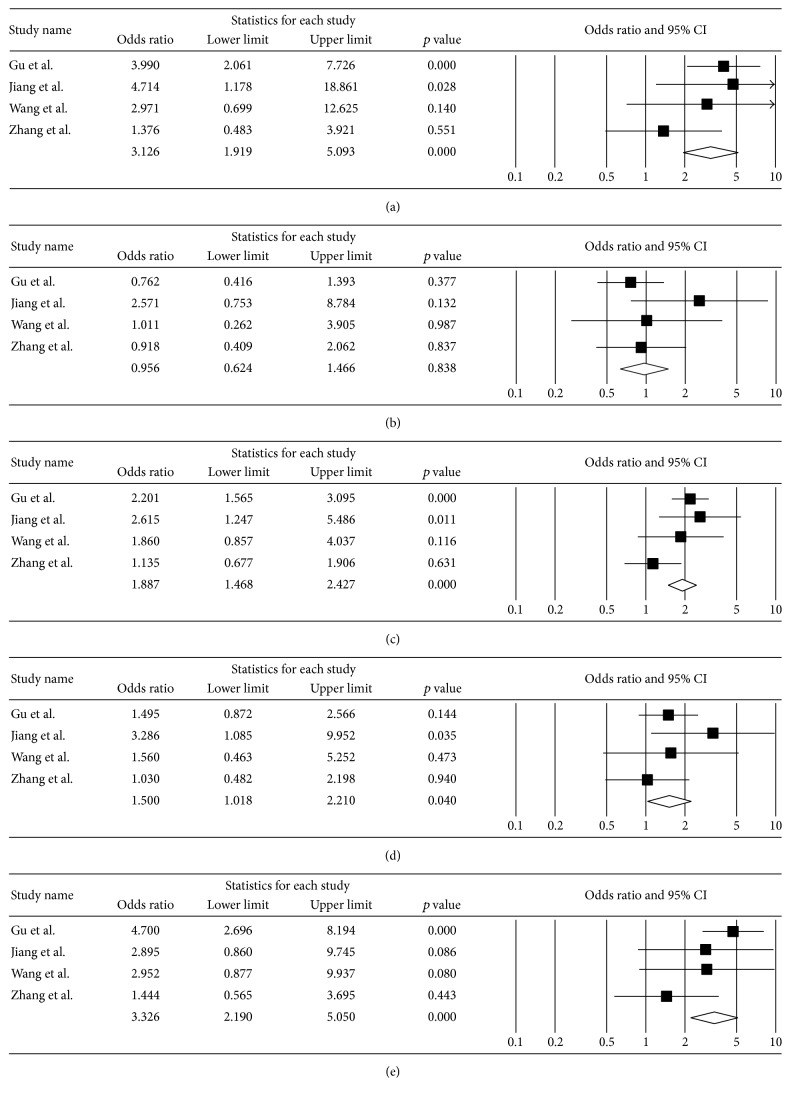
Odds ratio and 95% CI of individual and pooled data for the* ACE* insertion/deletion polymorphism and susceptibility to COPD in Asian. (a) D/D genotype versus I/I genotype, (b) I/D genotype versus I/I genotype, (c) D allele versus I allele, (d) D/D genotype + I/D genotype versus I/I genotype, and (e) D/D genotype versus I/D genotype + I/I genotype.

We performed a meta-analysis in two subgroups based on ethnicity: Asian population and Caucasian population. Results of the analysis in the Asian subgroup and the Caucasian subgroup are presented in Table 2 and Figure 1. For the dominant model (D/D genotype + I/D genotype versus I/I genotype), OR was 1.500 (95% CI = 1.018–2.210, *p* = 0.040) and *p* value and *I* ^2^ for heterogeneity were 0.412 and < 0.001, respectively, in the fixed model. For the recessive model (D/D genotype versus I/D genotype + I/I genotype), OR was 3.326 (95% CI = 2.190–5.050, *p* < 0.001) and *p* value and *I* ^2^ for heterogeneity were 0.203 and 3.835, respectively, in the fixed model. OR for the homozygote comparison (D/D genotype versus I/I genotype) was 3.126 (95% CI = 1.919–5.093, *p* < 0.001) and *p* value and *I* ^2^ for heterogeneity were 0.358 and 6.964, respectively, in the fixed model. In the allele model, D allele of the ACE gene showed a significant association with the risk of COPD (OR = 1.887, 95% CI = 1.468–2.427, *p* < 0.001).

The present results obtained from these genetic models suggested that insertion/deletion polymorphism of the* ACE* gene was significantly associated with a strong risk of COPD in the Asian population but not in the Caucasian population. Figures 1(a)–1(e) show the meta-analysis results for the codominant, dominant, and recessive models and the allele model in the Asian population, respectively.

To identify publication bias in the meta-analysis, funnel plots for all comparison models were generated to detect the presence of publication biases. The shapes of all of the funnels were symmetrical. We also evaluated publication bias using Egger's regression. There was no publication bias (*p* > 0.05, Table 2).

## 4. Discussion

A high amount of ACE is located in the lung capillaries [36] and it is involved in catalyzing the conversion of angiotensin I and converts it to an active peptide, angiotensin II. Angiotensin II functions as a strong vasoconstrictor agent and controls pulmonary vascular tone [37].* ACE* insertion/deletion polymorphism is associated with serum ACE levels [20] and D allele is related to increased formation of angiotensin II [38]. Thus,* ACE* insertion/deletion polymorphism has been reported to have an association with various diseases. The relationships between* ACE* polymorphism and autism [39], the pathogenesis of pulmonary hypertension [40], psoriasis [41], chronic kidney failure [42], and so on have been reported. As mentioned above, the deletion polymorphism, the D allele, of the* ACE* gene is associated with a higher ACE activity [20], and an elevation of the ACE activity was observed in COPD patients [43]. Therefore, the associations between* ACE* polymorphism and COPD have been investigated in many previous studies [13–16, 26, 28, 31, 44, 45]. However, the results of previous studies regarding the association between* ACE* polymorphism and COPD were conflicting and contradictory.

Previous two meta-analysis studies that assessed the relationship between* ACE* polymorphism and COPD risk failed to verify the link in the Caucasian population, but they found an association in the Asian population [46, 47]. Our meta-analysis results were in agreement with the previous results. A total of 977 COPD cases and 1,092 controls were included in the present study. We could not identify any statistically significant association between* ACE* polymorphism and COPD risk. But when we performed a meta-analysis in the subgroup based on ethnicity, we found a strong association between* ACE* polymorphism and COPD risk in the Asian population (*p* < 0.001) but not in the Caucasian population. A previous study examined the ratio of the frequencies of* ACE* II, ID, and DD genotypes among various ethnicities. The study showed that the ratio of the frequencies of* ACE* II, ID, and DD genotypes was 1 : 2 : 1 in the Europeans; there was a tendency towards a higher D allele frequency in the Nigerians, and there was a tendency towards a higher I allele frequency in the Samoans and the Yanomami Indians [48]. Although our study did not include Nigerians, Samoans, or Yanomami Indians, allele distribution between the Asian and Caucasian populations was different (all studies in the Asian population showed a higher I allele frequency). This might have led to a difference in the results between the two ethnicities.

According to GBD study, age-standardized death rates for COPD are higher in low-income countries such as South Asian and Sub-Saharan African countries [49]. But, the rate is similar in developed countries. In Korea, medical costs per person were US$ 2,803 ± 3,865 in 2009 and the total cost of COPD-related medications increased by 33.1% over 5 years [50]. Also, patients with high grade COPD impose three times higher economic burden ($3,744 versus $1,183) on the health care system in Korea [51]. In Singapore, the mean total cost was approximately $9.9 million per year and 42% of the total cost burden was incurred for the medical management of COPD [52]. Thus, the importance of prediction and early diagnosis of COPD, both clinically and economically, has increased.

In the present study, we collected previous studies that assessed the relationship between COPD risk and* ACE* polymorphism, but our study has some limitations. Our results showed the association between* ACE* polymorphism and COPD risk. However, we could not investigate various ethnic distributions because most of the studies included only the Caucasian and Asian populations. Also, environmental factors are key factors in COPD, but we could not consider the environmental factors in this meta-analysis. As mentioned above, our results showed no evidence of publication bias, but some results were influenced by the included articles.

Despite some limitations, our results showed a statistically significant difference in the allele and genotype distribution in the Asian population. If more results in various populations are obtained in further studies, the relationship between* ACE* polymorphism and the development of COPD can be clarified.

## Figures and Tables

**Table 1:** Information of eligible studies included in the meta-analysis.

Author	Year	Country	COPD/control	COPD			Control			COPD		Control		HWE in control
				I/I	I/D	D/D	I/I	I/D	D/D	I	D	I	D	
Simsek	2013	Turkey	66/40	15	20	31	8	19	13	50	82	35	45	0.823
Ulasli	2013	Turkey	80/49	16	17	49	6	10	33	49	111	22	76	**0.004**
Ayada	2014	Turkey	47/64	8	26	13	8	28	28	42	52	44	84	0.808
Ahsan	2004	India	27/66	10	12	5	23	33	10	32	22	79	53	0.743
Gu	2003	China	122/159	28	37	57	49	85	25	93	151	183	135	0.235
Hopkinson	2008	UK	103/101	29	49	25	28	49	24	107	99	105	97	0.777
Jiang	2004	China	30/30	7	12	11	15	10	5	26	34	40	20	0.171
Pabst	2009	German	152/158	43	76	33	39	69	50	162	142	147	169	0.124
Tkáčová	2005	Slovakia	66/118	15	31	20	19	68	31	61	71	106	130	0.074
van Suylen	1999	Netherlands	87/95	17	43	27	17	50	28	77	97	84	106	0.514
Wang	2000	China	20/38	5	7	8	13	18	7	17	23	44	32	0.861
Yildiz	2003	Turkey	42/40	7	21	14	10	18	12	35	49	38	42	0.536
Zhang	2008	China	61/57	21	27	13	20	28	9	69	53	68	46	0.877
Busquets	2007	Spain	74/77	7	40	27	11	53	13	54	94	75	79	**<0.001**

COPD: chronic obstructive pulmonary disease; ACE: angiotensin-converting enzyme; I/I: insertion/insertion genotype; I/D: insertion/deletion genotype; D/D: deletion/deletion genotype; HWE: Hardy-Weinberg equilibrium.

**Table 2:** Overall analysis between *ACE* insertion/deletionpolymorphism and susceptibility to COPD.

Comparisons	Population	Heterogeneity		Model	OR	95% CI	*p*	Egger' *p*
		*p*	*I* ^2^					
D/D versus I/I	All	0.005	58.855	Random	1.299	0.840−2.010	0.240	0.612
	Asian	0.358	6.964	Fixed	3.126	1.919−5.093	**<0.001**	0.740
	Caucasian	0.731	<0.001	Fixed	0.852	0.619−1.172	0.325	0.209
I/D versus I/I	All	0.846	<0.001	Fixed	0.904	0.714−1.145	0.404	0.368
	Asian	0.384	1.692	Fixed	0.956	0.624−1.466	0.838	0.249
	Caucasian	0.862	<0.001	Fixed	0.882	0.664−1.172	0.386	0.800
D versus I	All	0.001	66.564	Random	1.167	0.915−1.489	0.213	0.511
	Asian	0.156	42.596	Fixed	1.887	1.468−2.427	**<0.001**	0.861
	Caucasian	0.574	<0.001	Fixed	0.926	0.790−1.086	0.347	0.222
D/D + I/D versus I/I	All	0.509	<0.001	Fixed	1.040	0.835−1.295	0.728	0.451
	Asian	0.412	<0.001	Fixed	1.500	1.018−2.210	**0.040 **	0.567
	Caucasian	0.941	<0.001	Fixed	0.874	0.670−1.141	0.323	0.602
D/D versus I/D + I/I	All	<0.001	72.535	Random	1.364	0.888−2.093	0.156	0.624
	Asian	0.203	3.835	Fixed	3.326	2.190−5.050	**<0.001**	0.347
	Caucasian	0.229	25.041	Random	0.955	0.710−1.286	0.764	0.278

COPD: chronic obstructive pulmonary disease; ACE: angiotensin-converting enzyme; OR: odds ratio; CI: confidence interval; I/I: insertion/insertion genotype; I/D: insertion/deletion genotype; D/D: deletion/deletion genotype.
